# Biosensing Applications Using Nanostructure-Based Localized Surface Plasmon Resonance Sensors

**DOI:** 10.3390/s21093191

**Published:** 2021-05-04

**Authors:** Dong Min Kim, Jong Seong Park, Seung-Woon Jung, Jinho Yeom, Seung Min Yoo

**Affiliations:** 1 Center for Applied Life Science, Hanbat National University, Daejeon 34158, Korea; dmk.iqbio@gmail.com; 2 School of Integrative Engineering, Chung-Ang University, Seoul 06974, Korea; toxiart@gmail.com (J.S.P.); bioboyandy@gmail.com (S.-W.J.); jinho0134@gmail.com (J.Y.)

**Keywords:** localized surface plasmon resonance, detection, nanoparticle, nanostructure, biomolecule

## Abstract

Localized surface plasmon resonance (LSPR)-based biosensors have recently garnered increasing attention due to their potential to allow label-free, portable, low-cost, and real-time monitoring of diverse analytes. Recent developments in this technology have focused on biochemical markers in clinical and environmental settings coupled with advances in nanostructure technology. Therefore, this review focuses on the recent advances in LSPR-based biosensor technology for the detection of diverse chemicals and biomolecules. Moreover, we also provide recent examples of sensing strategies based on diverse nanostructure platforms, in addition to their advantages and limitations. Finally, this review discusses potential strategies for the development of biosensors with enhanced sensing performance.

## 1. Introduction

Biosensors are analytical devices that consist of biological recognition elements, transducer components, and electronic systems. These devices are typically used to detect and monitor chemicals and biomolecules in clinical and environmental settings. In particular, point-of-care (POC) testing and monitoring has become an ongoing trend for the development of medical and environmental applications due to the increasing need for user (patient)-centered systems, and more convenient, inexpensive, portable, and accurate detection systems that can be easily deployed in a wide variety of scenarios [1]. A recent report by the World Health Organization (WHO) suggested that the ideal analytical method should be affordable, sensitive, specific, user-friendly, rapid and robust, equipment-free, and deliverable to end-users. These criteria, represented by the acronym ASSURED, provide a framework for the evaluation of detection devices, especially in resource-limited regions or field applications. Biosensor development entails not only the fabrication of detection devices but also evaluating their sensing performance. Fabrication often involves the selection of a substrate and receptor, followed by functionalization of the substrate with the receptor. The sensing performance of the newly-developed device is then validated by analyzing the signals generated by the chemical reaction between the receptor and a target analyte (e.g., pathogenic microorganisms or their nucleic acids). Importantly, the fabrication and evaluation phases provide a crucial basis for the improvement of signal intensity and accuracy.

Biosensors are typically classified based on transducer type as optical, thermal, piezoelectric, quartz crystal microbalance, and electrochemical, among others. Among these, optical biosensors allow for practical, rapid, portable, and cost-effective detection and monitoring of diverse chemicals and biomolecules [1]. Further, plasmonic sensors, a type of optical sensors, offer high sensitivity and multiplexing capability and do not require relatively expensive proprietary instruments. Therefore, several types of plasmonic sensors have been developed to date, including surface plasmon resonance (SPR) [2], long-range surface plasmon polariton (LRSPP) [3], surface-enhanced Raman scattering (SERS) [4,5], and localized surface plasmon resonance (LSPR) [1,6,7] sensors. LSPR biosensors offer several advantages over other plasmonic sensors, including their inherent label-free nature, portability, low cost, and real-time sensing capacity. Additionally, nanostructure platform-based LSPR biosensors are under active commercial development, and some have even been commercially launched by several companies such as LSPR AG Co., LamdaGen Co., and others. Therefore, it is expected that LSPR sensors will have increasingly important roles in POC testing and monitoring in both clinical and environmental settings.

There are many good reviews that provide detailed information on the principles and characteristics of LSPR technology [8,9,10,11,12]. In this article, focus is given to the biomolecule-detecting strategies and technologies based in diverse nanostructure platforms, which can be divided into three different categories: solution phase-based colloidal nanoparticles, flat substrate-based platforms, and nanoparticle (NP)-coated optical fiber platforms. Representative applications of these strategies, including those described in this paper and others that have been developed and employed for the detection of a wide range of chemical and biomolecules, are also described in Table 1. Discussion of potential challenges and strategies to improve the performance of LSPR biosensors, summarized in Table 2, will be helpful to all researchers in the development of LSPR sensors for biosensing applications. An approach that may enable the development of biosensors with an enhanced sensing performance for POC detection is described to help achieve the long-term goal of its practical use.

## 2. LSPR Biosensors for POC Molecule Detection and Monitoring

LSPR is a phenomenon that occurs when the electromagnetic radiation of incident light interacts with the free electrons of metal nanostructures. Upon light incidence, resonance occurs if the frequency of the collective electron oscillations matches that of the conduction band of the metal, after which most of the light energy is transferred to free electrons. When surface waves occur, this phenomenon is referred to as surface plasmon resonance (SPR). Linear polarized free-space incident light generates an electric field at the plasmonic surface, after which light-induced surface plasmon polariton (SPP) propagates along the surface, thereby reducing spatial resolution. However, when light is irradiated onto plasmonic nanoparticles or nanostructured surfaces, the SPP is confined to the surrounding nanoparticles or nanostructures. This strong enhancement of the local electromagnetic fields generated at the nanostructure level occurs due to the frequency-matched collective oscillation of electrons in the conduction band of the metal. The intensity of the generated electromagnetic field is dramatically enhanced in these circumstances but decreases sharply as the distance from the surface increases [13]. Therefore, LSPR refers specifically to non-propagating plasmon modes. LSPR biosensors take advantage of the aforementioned plasmonic properties of noble metals and strong electromagnetic fields created upon plasmon resonance around confined structures. Therefore, nanostructure is an important factor that affects biosensor sensitivity. Resonance depends on the size, shape, material, composition, surface modification, interparticle spacing, dielectric environment, and separation distance of the conductive band [14,15,16,17]. Variations in nanostructure or nanoparticles with separations of a few nanometers can further amplify the amplitude of the confined electromagnetic field. An enhanced electromagnetic field intensity can allow for the sensitive detection (e.g., single-molecule detection) of molecules bound to the surface or to nanoparticles [13,18]. The binding of analytes on surfaces leads to a change in the refractive index of the surrounding medium such as the metallic surface, which can be identified as a wavelength shift or a change in LSPR peak intensity.

Many efforts have been made to develop LSPR-based sensors for clinical and environmental applications, because LSPR sensing approaches offer many advantages in POC monitoring and point of view detection. More importantly, LSPR-based sensors enable on-site measurement. This is largely because this technology does not require modifications or further labeling of analytes, thus enabling simple and rapid detection of target molecules. This label-free detection is possible because changes in intensity and wavelength shifts occur solely via the interaction between the analyte in the samples and the receptor immobilized on the nanostructure without the need for any conjugating reporter molecules. Moreover, this procedure is not affected by temperature or sample composition, among other factors [13,19]. In particular, this type of sensor is suitable for the detection of molecules in biological samples and clinical specimens because these sensors are unaffected by ionic properties [20]. The stability of LSPR in response to environmental changes is due to its compact sensing volume and short electromagnetic field decay length, resulting in a higher sensitivity to molecular binding and lower sensitivity to bulk refractive index changes. Moreover, given that LSPR focuses on a spectral shift and not an angle change, it is much more resistant to environmental vibration and mechanical noise than other similar approaches [21]. The simple design and portability of LSPR instruments are additional advantages of POC sensors. In particular, LSPR sensors do not require prisms to refract the incident light and therefore LSPR-based optical hardware is much less complex, smaller, and more affordable. As mentioned previously, given that LSPR is less susceptible to temperature changes and does not require strict temperature control, LSPR instruments can be much simpler and easier to use and maintain. Therefore, the applications of LSPR sensors could soon expand to food, beverage, and drinking water safety testing, in addition to disease diagnostics. The following sections highlight relevant LSPR biosensors that have been applied for biomolecule and chemical detection in clinical analyses and environmental monitoring.

The molecule-detecting performance of LSPR sensors largely depends on the distribution and concentration of hot spots. These hot spots are confined locations in close proximity to nanostructures in which the highest electromagnetic field strengths occur. A high abundance of hot spots on the nanostructure leads to a large change in the resonance wavelength shifts caused by analytes adsorbed on the surface, thereby enhancing the sensitivity of the sensor. Regular hot spots generated on uniform nanostructures result in sharp and clean resonance spectra, thus providing a reliable and reproducible result. The structures used for LSPR sensors can be classified into three different categories: (1) solution phase-based colloidal nanoparticles, (2) flat substrate-based platforms, and (3) NP-coated optical fiber platforms.

## 3. Current LSPR Biosensors for the Detection of Chemical and Biomolecules

Challenges and strategies to improve the performance of LSPR biosensors, and the related parameters (linear range, LoD, assay time, etc.) are summarized in Table 1 and Table 2, respectively.

### 3.1. LSPR Sensors Coupled with Solution Phase-Based Nanoparticles

The use of solution phased nanoparticles can be advantageous for the fabrication of substrates, because the nanoparticles themselves can act as an LSPR substrate without additional processing requirements. Solution-based biosensors generally use colloidal nanoparticles and non-spherical shapes such as rods and spheres, which are commonly used as LSPR substrates. The aggregation or self-assembly of nanoparticles, nanorods, and nanowires results in randomly distributed nanostructured colloids. In these structures, hot spots can be generated at the poles of the nanospheres near areas of higher surface curvatures, gaps, or junctions, resulting in a high refractive index change sensitivity [3,22,23,24]. Advances in nanoparticle synthesis and nanofabrication techniques have also allowed for the development of metallic nanostructures. Metal nanoparticles with specific geometries such as nanoprisms [25,26] exhibit higher electromagnetic field strengths than isotropic nanoparticles due to the accumulation of oscillating charges on the edges of the anisotropic nanoparticles. Additionally, nanostructures can be formed from self-assembled nanoparticles linked through functionalization with molecules, where the molecule size determines the distance of the gap between the nanostructures. Therefore, different molecules will result in gap variations, resulting in enhanced hot spot effects [27,28,29]. Self-assembled nanoparticles can also form randomly misaligned structures, where edge-to-edge nanoparticles (i.e., gaps) induce a double resonance effect [30]. Importantly, nanoparticle length misalignment and thickness can determine double resonance strength and position. These optical properties may therefore contribute to the development of nanoswitches, nanomotors, nanorulers, and dual-channel biosensors [25].

**Figure 1:**
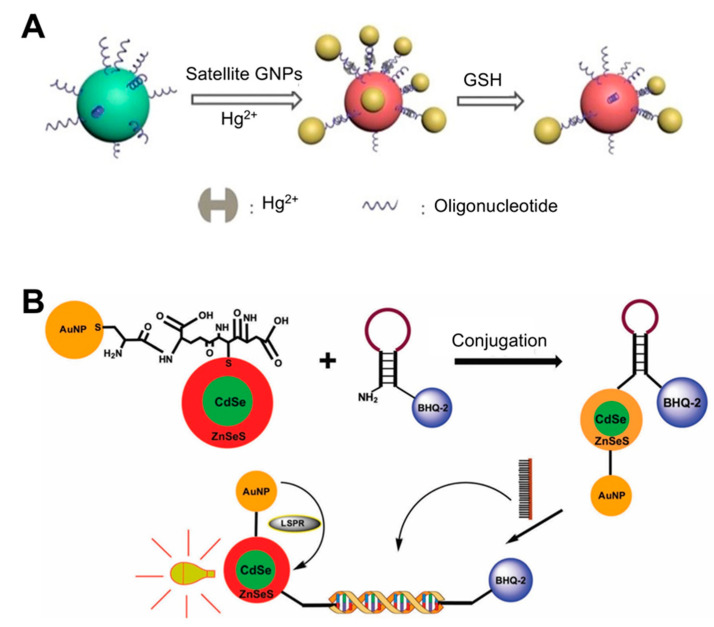
LSPR biosensing strategies based on solution phase-based nanoparticles: (**A**) Glutathione (GSH) detection using Au core-satellite structures containing Hg^2+^. Reproduced with permission from [36]; (**B**) Detection of dengue virus using CdSe/ZnSeS core/alloyed shell quantum dots via band-gap engineering. Reproduced with permission from [7]. GNP, gold nanoparticle.

The compositions of the nanoparticles used as LSPR substrates are additional factors that determine sensitivity. Several types of metal-based (e.g., europium, gold, silver, and copper) and non-metal-based (e.g., graphene, silica, and carbon nanotubes) nanoparticles have been used in biosensing to monitor a variety of biological analytes [31]. Conventional LSPR biosensors typically use coinage metal (e.g., Au and Ag) nanoshells, because they can support NP plasmon resonances in the ultraviolet, visible, and near-infrared regions of the spectrum, and are modifiable by adjusting the NP size and shape. AuNPs have a wide light absorption and scattering cross-section in the SPR wavelength regions. Due to their LSPR properties, self-assembly technology has led to the diversification of sensing structures, which now include gold dimers, trimmers, core-satellites, and clusters, among others [32,33,34,35], all of which exhibit plasmon coupling effects. Moreover, many methods involving oligonucleotide-AuNP have been widely adopted. For instance, a recent study reported the use of Au core-satellite structures containing Hg^2+^ for the detection of glutathione (GSH) ([36]; Figure 1A). This structure was first synthesized by assembling different-sized AuNPs (50 nm NPs for the core and 13 nm NP for satellites), which consisted of functionalized DNA strands with complementary sequences that acted as linkers bridging two different AuNPs via base pairs. To endow this structure with the ability to detect GSH, the authors took advantage of the distinct capacity of GSH to bind to specific heavy metal ions such as Hg^2+^, Cd^2+^, and Pb^2+^. Therefore, Hg^2+^ was added to the structure by incorporating it into the DNA duplex via T-Hg^2+^-T base pairing. When GSH is present in the sample, the higher affinity of GSH and Hg^2+^ than that of T-Hg^2+^-T leads to a structural collapse, resulting in a strong blue shift in the LSPR peak (0.1 μM detection limit) within 30 min.

Some studies have conjugated AuNPs and other materials to improve sensing performance. For example, the conjugation of AuNPs and quantum dots (Qdots) results in an intense and narrow luminescence emission, photostability, low toxicity, and broad absorption wavelengths. When Qdots are conjugated with AuNPs, the LSPR from the surfaces of the AuNPs enhances the fluorescence intensity of the Qdots and therefore photoluminescence (PL) emission increases [37,38,39]. Using these characteristics, a biosensor for four different serotypes of dengue virus was developed using CdSe/ZnSeS core/alloyed shell Qdots via band-gap engineering to generate specular optical properties ([7]; Figure 1B). This band-gap engineering approach was achieved via passivation with a ternary alloyed shell layer. To detect the viral DNA, the authors employed a molecular beacon (MB) containing a sequence that was complementary to the target DNA whose ends were attached with BHQ-2 as a quencher and AuNP-Qdot as a fluorophore reporter. If a given sample did not have a target DNA, the MB would continue forming a stem-loop, where BHQ-2 quenches the LSPR fluorescence of the AuNP-Qdot, because these molecules were in close proximity. When target DNA is present in the sample, the MB can hybridize with the DNA, thereby undergoing conformational changes and emitting a PL signal. Using this system, the dengue virus was detected with a detection limit of 20 copies/mL.

Sensors that employ salt-induced AuNP aggregation often undergo non-specific (i.e., unintended) absorption of analytes, inhibiting salt aggregation and lowering the accuracy of the sensor. For example, aromatic compounds such as ochratoxin A (OTA), adenosine triphosphate (ATP), and 17β-estradiol have an affinity toward Au and therefore this interaction prevents salt-induced AuNP aggregation. If OTA is mixed with ochratoxin-specific aptamer-bound AuNPs, aptamers prefer to bind to OTA, which results in a salt-induced NP aggregation and a purple coloration. However, at high OTA concentrations, OTA binds to both the AuNP surface and the aptamer, and therefore the lack of AuNP aggregation is directly caused by OTA and not by the aptamer. To accurately quantify OTA, Liu et al. used a double calibration curve method [40], where different calibration curves were adopted depending on the OTA concentration. Using this method, OTA could be quantitatively detected in 15 min within a 0.0316–310 ng/mL range. Despite many advances in the solution phase NP-based strategy, additional efforts are continuously required to accurately and consistently control the size and shape of NPs, both of which directly affect the sensitivity, specificity, and reproducibility of sensing procedures.

### 3.2. LSPR Sensors Using Flat Substrate-Based Platforms

Compared to colloidal nanoparticles, flat substrate-based LPSR sensors provide higher reproducibility and allow for the detection of multiple targets. These sensors largely fall within two sensing platforms: uniformly distributed NPs on a substrate and periodic nanopatterns.

Deposition of nanomaterials on flat substrates can be achieved via annealing and deposition procedures using focused ion beams, holographic lithography, nanosphere lithography, elastic soft lithography, and template stripping [41,42,43]. Using this approach, diverse nanostructures, such as nanoshells, dimer/trimer nanoantennas, nanostars, nanocrescents, and nanopyramid arrays have been produced and deposited on diverse flat substrates [44,45,46,47,48,49]. Periodic nanopatterns can be fabricated using electron beam lithography or mask-assisted techniques, which render regular patterns such as nanoholes, nanofillars, and nanodisks [50,51,52,53,54]. Flat substrate-based LSPR sensors facilitate data acquisition because they feature a high signal-to-noise ratio and thus a high sensitivity compared to LSPR sensing systems based on isolated NPs [55,56,57]. However, their production entails multiple steps and high costs [58].

**Figure 2:**
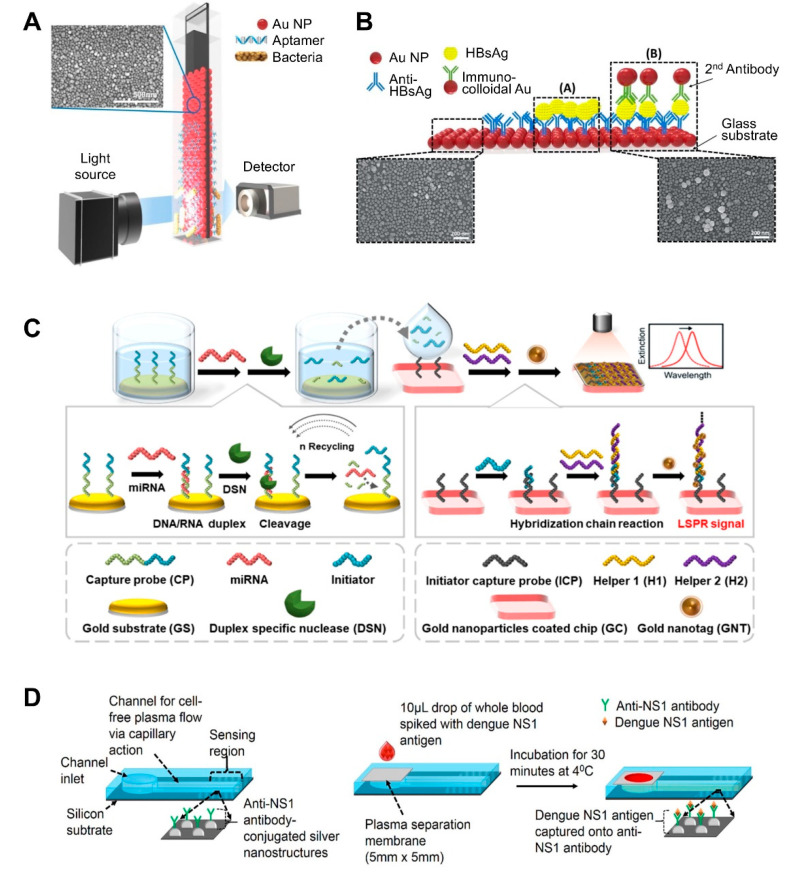
LSPR biosensing strategies based on nanoparticle (NP)-deposited flat substrates: (**A**) Detection of bacterial cells using aptamer-functionalized Au-coated glass slides. Reproduced with permission from [57]; (**B**) Detection of hepatitis B surface antigen (HBsAg) using a heteroassembled sandwich structure on a glass slide consisting of antibody-conjugated AuNPs and anti-HBsAg-conjugated AuNPs. Reproduced with permission from reference [59]; (**C**) Detection of miRNA using a duplex-specific nuclease-mediated target recycling reaction-coupled LSPR sensor on a Au substrate. Reproduced with permission from [63]; (**D**) Detection of the NS1 antigen of the dengue virus in whole blood using a silver NP-deposited silicon substrate functionalized with antibodies, which was coupled with a polyethersulfone membrane filter at the inlet of the biosensor for plasma separation. Reproduced with permission from [65].

Most flat substrate-based LSPR sensors use glass as a flat substrate on which to deposit AuNPs [31,57,59,60,61,62,63,64]. One of the simplest ways to fabricate such LSPR sensing substrates is to dip glass coated with 0.5% 3-aminopropyltriethoxysilane (APTES) as an amine group linker into an AuNP solution ([57]; Figure 2A). Once uniformly coated with AuNPs, the substrate (glass) exhibits a high absorbance signal. To detect bacterial cells using this substrate, Oh et al. immobilized thiolated species-specific aptamers on the substrate and incubated with whole cells, including *Salmonella typhimurium*, *Lactobacillus acidophilus*, and *Pseudomonas aeruginosa*. This sensor achieved a 10^4^ CFU/mL limit of detection within 30–35 min. They further optimized the production conditions for AuNP-deposited glass substrates [60]. Specifically, the authors investigated the effect of glass thickness and quality on the properties of the final product. All substrates exhibited a uniform and dense AuNP deposition layer. To achieve this, the glass was treated with 0.5% of APTES at 60 °C and then incubated with an AuNP solution for 8 h. To detect C-reactive protein (CRP) using this substrate, the substrate was additionally incubated with anti-CRP antibody, resulting in an 11.28 ng/mL limit of detection with a 0.01–10 μg/mL quantifiable range.

Au nanorod (GNR) was also deposited on the glass and applied to detect various biomolecules [61,62]. To improve the LSPR signal of the GNR substrate, a signal enhancer molecule (i.e., berberine) was used for improving the LSPR signal shift [61]. Berberine makes the LSPR signal more red-shifted by intercalating itself into the G-quadruplex structure formed when the aptamer binds to the analyte and suffers a conformational change, resulting in enhanced sensitivity and broader dynamic range compared to berberine-free sensors. This sensor could detect OTA, aflatoxin B1, ATP, and K^+^ at sensitivities of 0.56, 0.63, 0.87, and 1.05 pM, respectively, accounting for a 1000-fold increase in the detection limit with a broad dynamic range of 10 pM to 10 μM with linearity. As another means to improve the LSPR signal, the surface of the GNRs was functionalized with citrate as a stabilizer [62]. The surface modification with citrate offered a good dispersion of the GNR particles without surfactant and high immobilization efficiency of GNR on substrate, resulting in a large peak shift. The improved LSPR sensor could detect 25-hydroxyvitamin D_3_ down to 0.1 ng/mL.

AuNPs can form multiple layers on flat substrate by sequentially binding to other different-sized NPs, which can enhance sensitivity. For example, heteroassembled sandwich structure-based LSPR sensors have been developed for the detection of hepatitis B surface antigen (HBsAg) ([59]; Figure 2B). This sensor substrate was prepared by depositing Ab-conjugated AuNPs on glass and then exposing it to HBsAg. Then, additional anti-HBsAg-conjugated AuNPs were added, thus forming a sandwich structure. This AuNP bilayer provided enhanced LSPR signals, and smaller immunocolloidal AuNPs rendered stronger LSPR signals, because smaller NPs result in more hot spots. This sensor could detect HBsAg down to 100 fg/mL in 15 min within a 10 ng/mL to 100 fg/mL linear range.

After the deposition of AuNPs on flat substrate, this platform-based sensing approach can be additionally combined with enzyme reaction-assisted signal amplification methods, which can also enhance their sensitivity ([63]; Figure 2C). In particular, the distinct catalytic activities and enzymatic activities of DNA-modifying enzymes, such as DNA polymerases, nicking endonuclease, exonucleases, and ligases, could be used to enhance biosensor sensitivity and specificity due to their specific recognition capabilities and characteristic functional mechanisms [64]. Ki et al. developed a miRNA-detecting LSPR biosensor that incorporated signal amplification using a duplex-specific nuclease (DSN) [63]. In this study, capture probe consists of two parts: the first part contained sequences complementary to specific miRNA to form a DNA:RNA duplex and the other contained sequences that acted as initiators in the following step. Capture probe was first immobilized on an Au flat surface. When specific miRNA was present in a sample, miRNA formed duplexes with the capture probe. Given that DSN can only digest the DNA portion of the DNA:RNA duplex, except for the ssDNA part, the miRNA can dissociate from the duplex and participate in another binding event with the surface-bound Au. This miRNA reaction continues in a cycle, which is the first factor that improves the sensitivity of the sensor. By comparison, the remaining ssDNA portion (i.e., the initiator) of the capture probe sequentially binds to helper probes immobilized on another Au surface, thus forming sandwich self-assemblies. This second factor also enhances the sensitivity of the sensor. The third sensitivity-enhancing factor was the use of tannic acid-capped AuNPs. Tannic acid is a gallol-rich hydrophilic polyphenolic compound, which can bind to the phosphate backbone of DNA via hydrogen bonding. The resulting initiator-helper DNA-AuNPs complex dramatically changed the LSPR peak and therefore this sensor exhibited a linear range from 5 pM to 10 nM with a 2.45 pM miRNA limit of detection.

The deposition of NPs with different sizes and shapes on substrates can be used to detect multiple biomolecules because these NPs have different optical properties. A shape-code biosensor was developed using three different NP types (AuNPs (50 nm diameter), short NRs (1.6 aspect ratio), and long NRs (3.6 aspect ratio)) and applied to detect three different Alzheimer’s disease biomarkers: amyloid beta (Aβ) 1–40, Aβ 1–42, and τ protein [31]. The NPs were functionalized with specific antibodies and could detect Aβ 1–40, Aβ 1–42, and τ protein down to 4.9 fM, 26 fM, and 23.6 fM, respectively.

A recent study also explored the use of silicon as a substrate. The use of silicon allows for the large-scale fabrication of single-use sensing units, after which the sensor can be retrieved via chemical treatment. For example, Austin Suthanthiraraj et al. fabricated a silver nanostructure by thermally annealing a thin silver film deposited onto a silicon substrate ([65]; Figure 2D). Its shape was spheroidal, and its size ranged between 20 and 80 nm, with spacing ranging from a few tens to a hundred nm. The authors applied this sensor to detect dengue NS1 antigen in whole blood samples. A PDMS channel was created by placing a PDMS slab and its shallow inlet onto an as-fabricated nanostructure. Excess red blood cells and albumin in whole blood was filtered through polyethersulfone membrane on channel inlet because they can often interfere with the detection of analytes in whole blood. Therefore, only plasma was retained when blood was injected, and the antigen was captured by the antibody-coated sensing region. This sensor achieved a ~9 nm/μg/mL limit of detection.

Large-scale or large-area potential applications of an LSPR-based device were also achieved using copper due to the low cost of this material [66]. One of major drawbacks of using the Cu, Cu oxidation, was dramatically reduced by treating the Cu substrate with acetic acid. This easy-to-fabricate sensor consisted of a Cu shell/silica NP core structure arranged on an Au surface. Moreover, it can be manufactured in various formats with high reproducibility and possesses stable optical extinction properties that respond to ambient conditions. This sensor could be potentially used for quantitative and multiplex sensing of target DNAs with a 10 fM (50 zmol) limit of detection, thus enabling pathogen detection from clinical isolates.

**Figure 3:**
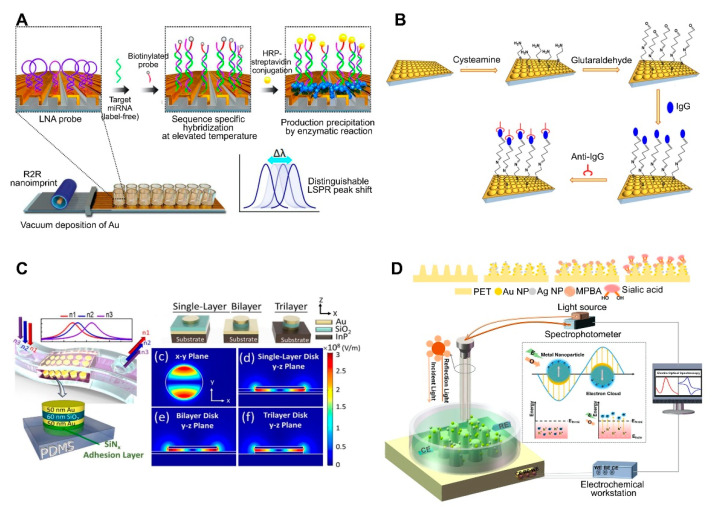
LSPR biosensing strategies based on nanopatterned structures: (**A**) Detection of miRNA using an inverted ‘L’-shaped nanostructure with nanograting pattern substrates functionalized with locked nucleic acids (LNAs). Reproduced with permission from [54]; (**B**) Detection of human IgG using a polystyrene (PS) sphere-deposited glass slide functionalized with antibodies, in which the use of PS of different sizes or shapes results in diverse cup substrates, enabling the modulation of plasmonic response. Reproduced with permission from [50]; (**C**) Detection of cancer cells attached to a metal–insulator–metal (MIM) integrative PDMS substrate. Reproduced with permission from [51]; (**D**) Detection of sialic acid by capturing with mercaptophenyl boronic acid (MPBA) via metal-S bond on nanocone shaped polyethylene terephthalate (PET) nanostructures densely covered with Au and AgNPs. Reproduced with permission from [67]. R2R, roll-to-roll; HRP, horseradish peroxidase.

The use of periodically ordered arrays is another method for large-area and low-cost sensor fabrication. This not only allows for the control of the size and shape of the sensor components but also provides a reproducible LSPR signal and strong three-dimensional (3D) local field amplification depending on surface geometry, resulting in an enhanced sensitivity. As mentioned above, the array pattern size, shape, and spacing affect the LSPR signal intensity. By taking advantage of the distinct size-, shape-, and spacing-dependent properties of these materials, periodically ordered arrays have been used for the fabrication of flexible, tunable, uniform 3D structure-based platforms [67,68,69,70,71,72]. For example, a recent study reported the synthesis of an inverted ‘L’-shaped nanostructure with a nanograting pattern consisting of an Au-deposited portion on the top and a PDMS layer on the sidewall surface ([54]; Figure 3A). The Au deposited top part was functionalized with a miRNA-detecting locked nucleic acid (LNA) that forms a hairpin structure. In the presence of miRNA and a biotinylated probe, the LNA hairpin structure stretches via base pairing, which is followed by horseradish peroxidase-streptavidin conjugate binding. Afterward, the enzyme-based precipitation of 4-chloronaphthol is induced by the conjugate, resulting in a peak shift of up to 37 nm. This sensor exhibited a miRNA detection limit of 13 fM.

Uniform and tunable platforms were fabricated using polystyrene spheres (PSs) as pattern generation tools ([50]; Figure 3B). PSs were deposited on glass on which PDMS was initially poured for substrate patterning. Afterward, the PDMS was peeled off to use as the sensor substrate, which was coated with a thin Au film to use the LSPR sensing platform. Different-sized PSs can be used to create PDMS-patterned plasmonic cups with different sizes and spacing between the cups, allowing for the modulation of the plasmonic response of the nanoplatforms, in addition to enhancing the 3D electromagnetic field. Therefore, this method provides a straightforward means to modulate the size/shape of nanostructures by simply changing the size/shape of the deposited nanoparticles, thus yielding nanoplatforms with diverse plasmonic responses. This platform-based sensor was applied to detect human IgG. Anti-human IgG antibodies were attached to an as-prepared platform, resulting in a 1.5 μg/mL limit of detection. Arrays of Au nanodisks with uniform and tunable structure were also fabricated on polymethylmethacrylate (PMMA) on glass substrate and applied to detect bacterial cells of *Staphylococcus aureus* in milk [72]. Varying diameters of Au disks could be achieved by using hole-mask colloidal lithography, in which PS was used as a mask to fabricate arrays of Au disks. Aptamer-functionalized arrays could detect whole *S. aureus* cells down to 10^3^ CFU/mL within 120 s without any pretreatment.

Recent advances in flat substrates-based LSPR sensors include the use of flexible nanostructures due to their practicality. More importantly, the main advantage of these sensors is their ability to deliver real-time and POC measurements. To achieve this, LSPR sensors have been developed by incorporating a metal–insulator–metal (MIM) integrative structure on PDMS. MIM structures are outstanding optical absorbers, because their wide angular range of incidence allows for the use of LSPR refractive index sensors under nonplanar surface conditions. A stretchable and highly sensitive detection device based on LSPR sensors with a MIM structure was recently reported. To fabricate this sensor, a MIM-disk LSPR sensor trilayer was first fabricated by sequentially depositing Au-SiO_2_-Au on an indium(III) phosphide substrate, then embedded onto PDMS, which acted as a transparent, biocompatible, and flexible substrate ([51]; Figure 3C). Enhanced sensitivity and PDMS absorption removal were achieved by changing the embedded depth of the MIM disk on the PDMS. This flexible MIM sensor exhibited a stable sensing performance on nonplanar substrates, with a sensitivity of 3.5 ± 1.75% depending on bending curvature.

Another recent trend in flat substrate-based LSPR sensor technology is to combine two different sensing approaches to enhance sensitivity. For example, electrochemical sensing was coupled with an LSPR system to detect sialic acid ([67]; Figure 3D). In this system, the nanostructures served both as electrodes for the electrochemical system and as the optical device for the LSPR sensing system. Electrochemical and LSPR signals are amplified via co-activation. Namely, electrochemical signals activate the electrons surrounding the AuNPs and AgNPs on a nanochip to promote surface plasmon propagation, whereas LSPR concentrates the electrons to enhance the current. The implemented nanostructure consisted of a nanocone-shaped flexible polyethylene terephthalate array, which was fabricated via laser interference lithography and reaction ion etching. The substrate was densely covered with AuNPs, and AgNPs and mercaptophenyl boronic acid were functionalized by capturing sialic acid via metal-S bonds. Using this sensor, sialic acid was detected at concentrations as low as 17 μM with a wide linear range of 0.05–5 mM.

### 3.3. Nanoparticle-Coated Optical Fiber-Based LSPR Sensors

LSPR technology allows for easy sample handling, does not require highly trained personnel, and can be implemented with small and inexpensive equipment, all of which favors its widespread adoption. The methods for the detection of specific analytes have been recently developed by dipping optical fiber into a sample to be tested [73,74,75]. The use of optical fibers as LSPR substrates has several advantages, including resistance to electromagnetic interference, chemical passivity, cost reduction, ease of use, and small footprint [76,77]. Easy reuse of the optical fiber-based LSPR sensor is also one advantage [75,78,79,80,81]. The general approach to reuse is to wash with phosphate buffered saline buffer or sodium dodecyl sulfate solution, and cut and polish the end of the used facet of the optical fiber. Such a simple reuse and recycling process of sensor would facilitate the practical and cost-effective sensing in diverse environmental setting, especially for regions with resource-limited settings. Notably, the combination of optical fiber and plasmonic nanostructures enables the development of miniaturized and portable sensing systems. Optical fiber is available in different configurations, including U-shaped, D-shaped, and tapered, among others [82,83,84].

As previously mentioned, in the LSPR sensor, the sensitivity is a major concern, and can depend on several factors such as the substrate sensing distance. Electric fields rapidly attenuate as the distance from the colloidal nanoparticles or the pattern substrates increases, resulting in generally short sensing distances of only a few tens of nanometers [13,21]. In optical fiber-based sensors, the amplitude of the resulting evanescent wave also falls exponentially as the distance from the fiber core increases because it uses light propagating through a dense fiber core through total internal reflection [85,86,87]. However, this limitation can be overcome by implementing an unclad core surface. The NP-modified fiber core and receptors mediate the evanescent wave interactions, thereby promoting the change in optical signals. Receptor-functionalized optical fiber/analyte binding events also occur, in which an LSPR peak shift can be induced [74]. Additionally, diverse strategies, such as resonant coupling of plasmons with nanoparticles or resonant molecules, precipitation reactions, and particle growth, have also been developed [88,89]. These strategies lead to additional changes in the refractive index of a surrounding nanostructure, resulting in an enhanced sensitivity. However, this approach requires additional operational steps and a secondary target or probe labeling.

**Figure 4:**
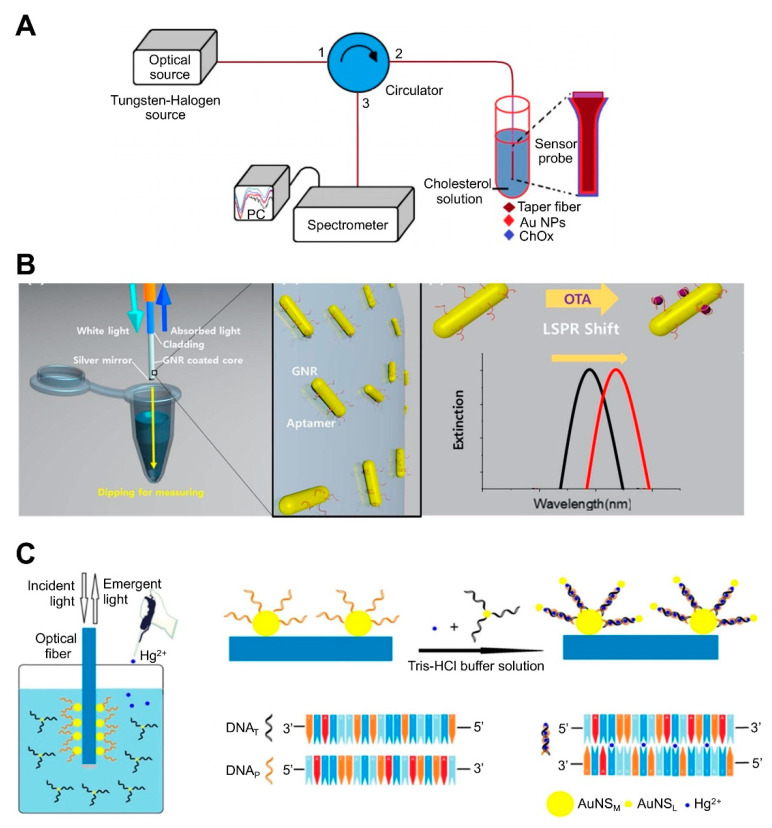
LSPR biosensing strategies based on nanoparticle (NP)-coated optical fibers: (**A**) Detection of cholesterol using cholesterol oxidase attached to an AuNP-immobilized fiber. Reproduced with permission from [85]; (**B**) Detection of ochratoxin A (OTA) using aptamer-modified gold nanorods (GNRs) immobilized on an optical fiber. Reproduced with permission from [74]; (**C**) Detection of Hg^2+^ using optical fiber functionalized with Au nanospheres (AuNS) and a thiolated DNA probe. Reproduced with permission from [75].

Optical fiber-based LSPR sensors have been developed to detect different chemical and biomolecules such as enzymes, cortisol, cancer markers, and bacterial cells [78,79,90,91,92,93]. For example, cholesterol was detected by attaching cholesterol oxidase (ChOx) to AuNP-coated fiber via an adhesive layer of (3-mercaptopropyl) trimethoxysilane. The ChOx enzyme specifically oxidizes cholesterol in the presence of oxygen, producing H_2_O_2_ and cholestenone. The level of H_2_O_2_ modulates the refractive index at the surface of the AuNPs and changes the reflectance intensity. AuNP-coated fiber has also been sequentially treated with MUA (carboxyl group)-EDC/NHS-ChOx ([85]; Figure 4A). Another study reported the detection of OTA using aptamer-modified GNRs immobilized on an optical fiber core surface ([74]; Figure 4B). For in situ detection of OTA based on optical fiber as the LSPR sensing platform, the sensing probe was dipped into a sample solution, after which the LSPR peak shift was monitored. OTA-specific aptamers were immobilized on GNRs, which were deposited onto thiol-functionalized glass. The specific binding of aptamer and OTA leads to a conformational change in the aptamer, thus increasing the local refractive index surrounding the GNR. This sensor could detect OTA concentrations as low as 12 pM with a 10 to 100 pM dynamic range. Another study developed an optical fiber-based sensor for the detection of Hg^2+^ ([75]; Figure 4C). The detection of Hg^2+^ relies on the T-Hg^2+^-T structure and plasmonic coupling effect. The sidewall of the LSPR fiber was firstly coated with an Au nanosphere monolayer (NSM) and then functionalized with a thiolated DNA probe (DNAP) via Au-S bonds. When the sample contained Hg^2+^, another DNA (DNAT) whose sequence was complementary to the probe DNA could bind with the probe via T-Hg^2+^-T base match, resulting in near field coupling enhancement. When the DNAT was attached to another small-sized Au NP (AuNSL), the DNAP:DNAT duplex induced a near field coupling effect due to the close proximity between AuNSM and AuNSL, which increases the refractive index at the AuNSM surface and causes a wavelength red shift, thus enhancing sensitivity. This sensor could detect Hg^2+^ at concentrations as low as 0.7 nM with a 1–50 nM dynamic range.

Recently, LSPR sensors coupled with different mode optical fibers were developed and applied to detect whole bacterial cells [78,91]. One example of these sensors exhibits two physical contact fiber structures: single mode fiber (SMF) and multi-core fiber (MCF). MCF structure exhibits high sensitivity to small RI variations due to intercoupling between cores of MCF and compact architecture. MCF has also low connection loss with SMF. This sensor was immobilized with DNA aptamer and could detect *Shigella sonnei* down to 1.56 CFU/mL within 5 min [91]. Another example is the tapered SMF–no core fiber–SMF structure. This fiber was functionalized with IgG antibody and could detect *S. aureus* with LoD of 3.1 CFU/mL [78].

## 4. Conclusions and Future Perspectives

Biosensing assays will become increasingly important in the near future, and will likely have a strong impact in the fields of clinical research, forensics, biodefense, food safety, animal health care, and pathology. However, the rapid, accurate, and multiplexed identification of biomarkers or causative agents in a variety of settings in a low-cost manner remains a major challenge of this field. The repeatability and reproducibility of sensing outcomes are also the important characteristics for measuring the precision of sensors. For repeatability, a range of values distributed relative to the actual value should be minimized at the same location, measurements, procedure, observer, and the measuring instrument within a short period of time. For reproducibility, the outputs from the experiments conducted by different individuals at different locations with different instruments should be consistent. There is currently an ever-increasing demand for disease diagnosis and environmental monitoring solutions, which highlights the need for further biosensing device development.

Future LSPR biosensors will likely incorporate novel nanomaterials, receptors, and sensing devices. Current research trends have established a departure from single-mode sensing devices in favor of multimodal biosensors, which incorporate a wide range of sensing approaches that are now being tested for molecule-detection applications [67]. These approaches have also been combined with microfluidic devices [51,61,94,95] and complementary metal–oxide–semiconductor (CMOS) devices [96,97] for the continuous and real-time detection of biomolecules. Additionally, new insights into the design and screening of synthetic receptors have been obtained using powerful selection methods such as systematic evolution of ligands by exponential enrichment (SELEX) and its alternatives [98,99,100], in addition to click chemistry [101,102], all of which will likely result in more sensitive and specific sensing of diverse biomarkers. These breakthroughs and innovations will undoubtedly lead to the emergence and discovery of new technologies and materials for disease diagnosis and environmental monitoring in the near future.

One important research topic regarding the detection of biomolecules is to improve the limit of detection without off-target effects. Modulating the LSPR frequency can affect sensitivity. Most LSPR biosensors focus on the use of metallic particles or nanostructures as a sensing platform; however, the metallic materials currently used are inherently limited. LSPR frequency control could be better implemented in semiconductor-based nanocrystals than in their metallic counterparts. Therefore, a wider range of biomolecules could be detected by broadening the sensing spectrum from the deep ultraviolet range (for the detection of nucleic acids and proteins) to the far infrared (for the detection of cells and tissues). A related study reported that the LSPR frequency varied from 1600 to 2200 nm by controlling the concentration of a tin dopant [103], and tungsten oxide nanoparticles exhibited an optical absorption from 900 to 1100 nm by doping additional oxygen atoms into their crystal structure to modify the carrier density [104]. However, additional efforts are required to discover novel plasmonic materials for the development of new platforms to detect diverse molecules.

Therefore, future studies should focus on the development of novel methods for surface modification and bio-conjugation on plasmonic materials. For instance, innovative functionalization methods can be achieved via click chemistry and using substrate-specific peptides or proteins. Click chemistry-based bioconjugation is a simple and fast procedure that enables process control and enhances chemo-selectivity [105]. Moreover, this approach does not perturb biomolecule activity due to the small size of its components [106]. For example, a silver enhancement signal amplification strategy can be employed to detect Cu(II) ions in aqueous solutions with high sensitivity. Based on a Cu(I)-catalyzed ‘click’ reaction between azide-functionalized AuNPs and an alkyne-modified glass slide, this method enables Cu(II) detection down to 62 pM. Combined with click chemistry, Qdots could be also used as fluorescent probes to visualize and track individual viral particles. Using this clickable surface modification strategy, multidentate–imidazole ligands were synthesized and living viruses could be attached to the Qdots. More importantly, the resulting virus-attached Qdots did not affect the viral infection [107]. Using the same strategy, Qdots were functionalized with avian influenza H5N1 pseudotype virus (H5N1p), and this conjugate exhibited bright and sustained fluorescent signals in mouse lung tissues, allowing for the noninvasive visualization of respiratory viral infections in real time [108]. Another surface modification strategy is to use substrate-specific peptides or proteins. Representative examples include metal-binding peptides, which act as a linker to modify the surface of metals such as Ni–Ti, Pt–W, Co–Cr, FeO, and Au [109,110,111]. To modify the metallic surface, these peptides or proteins must have a high affinity for metal, which affects the surface density of the grafted polymers, in addition to the biosensor sensitivity. The small size of these peptides or protein motifs allows them to form compact surface coatings, and therefore the desired function is minimally disrupted. Biocompatibility could also be an important factor to improve the surface properties of biosensors, particularly for medical purposes. Such peptides or protein motifs can be designed and selected using various methods such as phage display or cell surface display technology [111]. Recent advances have led to the development of machine learning-based computational prediction and computational approaches, which allow for the de novo design and redesign of metal-binding sites on proteins [112,113]. These strategies will surely enable the fabrication of surface platforms consisting of diverse plasmonic materials with specific and sensitive sensing performance, and the development of controlled and tunable LSPR technology. In addition, for commercialization of LSPR sensors, many practical assets, such as miniaturization, flexibility, and remote control, are desired.

**Table 1:** Examples of nanostructure-based LSPR biosensors for the detection of various molecules ^a^.

Classification	Substrate	Receptor	Analyte	Linear Range, LOD	Assay Time	Real Sample	Features	Reference
Solution phase-based nanoparticle	AuNP-based core-satellite structure	Hg^2+^ incorporating DNA duplex	Glutathione (GSH)	0.1 μM	30 min	ND	Use of property of GSH with high affinity for Hg^2+^. Caused a blue shift in the LSPR peak by AuNP structural collapse upon exposure to GSH.	[36]; Figure 1A
	CdSe/ZnSeS core/alloyed shell Quantum dot (Qdot)	DNA (molecular beacon)	Dengue virus	20 copies per mL	ND	ND	Quencher use: Change in PL Qdot depending on the presence/absence of target DNA in the sample. Conjugation of Qdots and AuNPs: boosting PL of Qdots by LSPR from AuNPs.	[7]; Figure 1B
	AuNP	None	Melamine	0 μM to 0.9 μM, 33 nM	ND	Liquid milk	Use of unmodified AuNPs without the need for a receptor due to the interaction of amine groups of melamine and AuNPs. Recovery rate of 99.2~111%.	[114]
	AuNP	Aptamer	Ochratoxin A (OTA)	0.0316–316 ng/mL	>15 min	Spiked corn	Use of color change based on AuNPs aggregation caused by competition between aptamer-bound Au NPs and OTA. Use of double calibration curve method to widen the detection range.	[40]
NP-deposited flat substrate	AuNP on the glass slide	Aptamer	*Salmonella typhimurium*	1.0 × 10^4^ CFU/mL, 10^4^ CFU/mL	>30–35 min	Pork meat	Fabrication of AuNP-coated transparent glass slide via a simple dipping adsorption method Use of APTES-immobilized glass slide to attach AuNPs	[57]; Figure 2A
Solid-based nanopatterned flatform	AuNP on the glass slide	Anti-CRP	C-reactive protein (CRP)	0.01–10 μg/mL, 11.28 ng/mL	ND	ND	Fabrication of a plasmonically active strip by depositing AuNPs on an APTES-immobilized glass slide. Use of cysteine-protein G to attach a receptor.	[60]
	Au nanorod (GNR)	Aptamer	25-hydroxyvitamin D_3_	0.1–10^5^ ng/mL, 0.1 ng/mL	ND	Human serum albumin sample	Use of citrate as a stabilizer of GNR: improving LSPR signal.	[62]
	Heteroassembled AuNPs	Antibody	Hepatitis B surface antigen	100 fg/mL–10 ng/mL, 10 pg/mL	>10–15 min	Human serum	Use of a multi-layered plasmonic structure by linking different-sized AuNPs.	[59]; Figure 2B
	GNR on glass slide	Aptamer	OTA, AFB1, ATP, and K^+^	0.56 pM for OTA, 0.63 pM for AFB1, 0.87 pM for ATP, 1.05 pM for K^+^	30 min	Ground corn powder, *Escherichia coli*, human serum	Use of berberine as an LSPR signal enhancer, which incorporates into the G-quadruplex structure that forms when the aptamer binds to the analyte and undergoes a conformational change.	[61]
	GNR on glass slide	Aptamer	Saxitoxin	5–10,000 μg/L, 2.46 μg/L	30 min	Mussel sample	Use of newly developed aptamers by implementing the graphene oxide (GOx)-SELEX method. Recovery rate of 96.13~116.05%.	[115]
	AuNP on glass slide	Antibody	Alzheimer’s disease biomarkers	4.9 fM for amyloid beta (Aβ)1–40,26 fM for Aβ1–42, and 23.6 fM for τ protein	ND	Human plasma	Multiplex detection using nanoparticles with different sizes and shapes, each of which was functionalized with various marker-specific antibodies.	[31]
	AuNP-coated glass slide	DNA	MicroRNAs (miRNAs)	5 pM to 10 nM, 2.45 pM	ND	Mouse Sample (urine and plasma)	Incorporation of LSPR signal amplification strategy using a duplex-specific nuclease-mediated target recycling reaction. Use of Au NP coated with tannic acid (a hydrophilic polyphenol compound) that can interact with the phosphate backbone of DNA, thereby enhancing LSPR signal.	[63]; Figure 2C
	Ag nanoprism on glass	DNA probe	Bacterial DNA	5 fg/μL of *E. coli* DNA, 300 cfu/mL	>15 min	ND	Fabrication of an LSPR platform by depositing Ag nanoprisms on poly-L-lysine-coated glass. Combined system consisting of microfluidic on-chip PCR and LSPRi using a digital micromirror device Real-time detection using a qPCR system.	[116]
	Ag nanocolumn on glass slide	Polymyxin B	Lipopolysaccharide endotoxin	340 pg/mL	ND	ND	Use of 3-mercaptopropionic acid to stabilize the Ag nanocolumn against oxidation and nanoparticle detachment in aqueous environments.	[117]
	Ag nanocolumn on glass slide	Antibody	Prostate-Specific Antigen	850 pg/mL	ND	ND	Use of 11-mercaptoundecanoic acid as a stabilizer of the Ag nanocolumn.	[118]
	Ag nanostructure on silicon substrate	NS1 antigen-specific antibody (IgG)	NS1 antigen of dengue virus	0.06 μg/mL	>30 min	Whole blood	Fabrication of nanostructures by E-beam evaporation and thermal annealing of thin silver film. Integration of polyethersulfone membrane filter at the inlet of a biosensor for plasma separation. Small sample volume requirements (10 μL of whole blood sample).	[65]; Figure 2D
	Nickel-doped graphene (NDG) on self-assembled gold nanoislands (SAM AuNI)	GOx	3-nitro-L-tyrosine (3-NT)	0.1 pg/mL–10 ng/mL, 0.13 pg/mL	ND	Human serum	Fabrication of imprinted nanostructure by thermal annealing of Au, followed by spin coating and thermal annealing of graphene and nickel. Use of strong energy adsorption by π–π stacking interaction between NO_2_ site of 3-NT and NDG	[119]
	Poly(mPD-co-ASA) on SAM-AuNI	Poly(m-phenylenediamine-co-ani-line-2-sulfonic acid) (Poly(mPD-co-ASA))	Pb^2+^	0.011 ppb–5 ppm, 0.011 ppb	ND	Drinking water	Use of poly(mPD-co-ASA) as a linker with AuNI and Pb^2+^ receptor.	[120]
	SAM-AuNIs	Anti-CD7 antibody	Exosome	0.194–100 μg/mL, 0.194 μg/ml	ND	Serum, urine	Use of exosome properties with high affinity for AuNI due to its negative zeta potential value	[121]
	SAM-AuNIs	Anti-IgG	Human IgG antigen	1 pM–100 pM, 1.188 pM	ND	Serum		[122]
	3D Au nanocups platform on polydimethylsiloxane (PDMS) surface	Antibody	Human IgG	1.5 μg/mL	ND	ND	Fabrication of imprinted nanostructures by deposition of a polystyrene (PS) monolayer on glass, pouring PDMS on a PS layer, peeling off the PDMS film, and coating the PDMS substrate with an Au film. Fabrication of uniform and tunable platform by changing the PS size.	[50]; Figure 3B
	Metal–insulator–metal (MIM) nanodisks on PDMS	None	Cancer cell (adherent cell)	NA	ND	ND	Construction of a MIM nanodisk consisting of Au-SiO_2_-Au on an InP substrate. Fabrication of a flexible sensor by transferring a MIM nanodisk onto PDMS.	[51]; Figure 3C
	Au and AgNPs on PET cone array structure	Mercaptophenyl boronic acid	Sialic acid	0.05–5 mM, 17 μM	ND	ND	Fabrication of core array nanostructures by depositing Au and AgNPs on (poly)ethylene terephthalate (PET). Combined system consisting of LSPR and an electrochemical sensing system.	[67]; Figure 3D
	Au-deposited 3D polyurethane acrylate (PUA) nanostructure	Locked nucleic acid	miRNAs	13 fM (2.6 attomole in 200 μL)	ND	Primary cancer cell lines	Fabrication of 3D plasmonic nanostructure consisting of roll-to-roll nanoimprint lithography-used PUA nanograting pattern, followed by Au deposition. Detection of miRNA single-base mismatches down to the attomole level by incorporating a biotin-streptavidin-horseradish precipitation reaction.	[54]; Figure 3A
	Au nano-ellipsoid array on quartz substrate	Anti-CD63 antibody	Exosome	1 ng/mL	<4 h	ND	Fabrication of nanostructures via AAO-templated Au deposition on a quartz substrate. Integration of LSPR and microfluidic systems.	[123]
	Au nanopillars on quartz coverslips	Anti-CD63 antibody	Exosome	ND	ND	MCF7 breastadenocarcinoma cells	Fabrication of Au nanopillar array by electron beam lithography. Enabled multiplexed measurement using LSPRi.	[52]
	Au nanopillar	Mercaptobenzoic acid	BSA	234 pM	ND	ND	Working in the visible and infrared region by changing the patterned shapes and interpillar distances.	[53]
	Au film on glass wafer	Anti-IgG, anti-TNF-α, anti-CRP antibody	IgG, TNF-α, CRP	10 ng/mL IgG, 10 ng/mL CRP	3.5 h	ND	Fabrication of nanostructure using physical vapor evaporation followed by a rapid thermal annealing treatment.	[124]
	Polymethylmethacrylate (PMMA) on glass substrate	Aptamer	*Staphylococcus aureus*	10^3^ CFU/mL	120 s	Milk	Fabrication of arrays of Au nanodisks on PMMA-treated glass substrate by using hole-mask colloidal lithography. Optimization of disk structure by varying diameter: improving LSPR signal.	[72]
NP-coated optic fiber-based platform	AuNPs-immobilized taperfiber	Cholesterol oxidase (ChOx)	Cholesterol	10 nM–1 µM, 53.1 nM	ND	ND	Fabrication of sensing component by sequentially coating MUA-EDC/NHS-ChOx on AuNPs-immobilized fiber.	[85]; Figure 4A
	GNRs immobilized on the optical fiber core surface	Aptamer	OTA	10 pM to 100 nM, 12.0 pM	ND	Grape juice	Detection by simply dipping an optical fiber into a sample solution, allowing in situ detection.	[74]; Figure 4B
	AuNPs-coated optical fiber	Anti-transferrin, protein A	Transferrin, protein IgG	ND	ND	ND	Combined system consisting of capillary LSPR sensors and metal–oxide–semiconductor image sensors. Use of AuNPs-coated capillary as a microfluidic channel and sensing surface. Multiple detection for high throughput screening of biomolecular interactions	[96]
	Optical fiber with copper oxide nanoflower (CuO-NF) and Au NPs-coated GOx structure	2-deoxy-D-glucose (2-DG)	Cancer cell	1 × 10^2^–1 × 10^6^ cells/mL, 2–10 cells/mL	ND	ND	Use of multi-core fiber structure. Coating of optical fiber with GOx and CuO-NF: increasing surface area and adsorption capability. Discrimination of cancer cells using 2-DG that binds to GULP receptor: the presence of more GULP receptors on cancer cell, inducing a peak shift. Reusable through washing with PBS.	[79]
	AuNPs-coated optical fiber	Aptamer	Zearalenone (ZEN)	1–480 ng/mL, 0.102 ng/mL	ND	Beer	Reusable by cutting and polishing a tip of optical fiber.	[80]
	Optical fiber	Anti-IgG antibody	IgG	1 fg/mL to 100 fg/mL, 7 aM	25–30 min	ND	Use of silver enhancer: amplifying the LSPR signal by catalytic reduction of silver around AuNPs. Use of U-bent optical fiber.	[90]
	Optical fiber	IgG antibody	*Staphylococcus aureus*	3.1 CFU/mL	ND	ND	Use of the tapered singlemode-no core-singlemode fiber coupler structure.	[78]
	MoS_2_/AuNPs-coated optical fiber	Aptamer	*Shigella sonnei*	1 – 1×10^9^, 1.56 CFU/mL	5 min	ND	Use of single mode fiber-multi-core fiber structure.	[91]
	AuPd alloy-coated plastic optical fiber	Anti-cortisol	Cortisol	1 pg/mL	ND	ND	Use of plastic optical fiber.	[92]
	Au film-coated optical fiber	Aptamer, HER2 antibody	Breast cancer HER2 protein	9.3 ng/mL (77.4 pM)	10 min	ND	HER2 biomarker detection using sandwich assay with anti-HER2 ssDNA aptamer and HER2 antibody.	[93]
	ZnO/AuNP-coated optical fiber	Ascorbate oxidase	Ascorbic acid	1 µM to 200 µM, 12.56 µM	ND	ND	Use of tapered optical fiber structure immobilized with ZnO-AuNPs.	[81]
	AuNP-modified the bare core	Probe DNA	Hg^2+^	1–50 nM, 0.7 nM	ND	Pond water	Functionalization of DNA-attached Au NP monolayer on optical fibers, resulting in an increase in the refractive index at the nanometer length region and near field coupling enhancement produced by close proximity to another-attached Au NPs via DNA-DNA hybridization Use of PAH, yielding enhanced sensitivity due to the higher density and less aggregation of Au NPs Reusability by dipping optical fiber into 1% SDS solution for 5 min	[75]; Figure 4C

^a^ Abbreviations: AuNP, gold nanoparticle; PL, photoluminescence; AFB, aflatoxin; ATP, adenosine triphosphate; CFU, colony forming unit; SELEX, systematic evolution of ligands by exponential enrichment; 3D, three-dimensional; AAO, anodic aluminum oxide; AuNI, gold nanoisland; LSPRi, LSPR imaging; PAH, poly(allylamine hydrochloride); SDS, sodium dodecyl sulfate; NP, nanoparticle; ND, Not determined.

**Table 2:** Challenges and strategies to improve the performance of LSPR biosensors for the detection of various molecules ^a^.

Challenge	Performance Improvement Strategies	Reference(s)
Sensitivity	Conjugation of Qdots	[7]
	NP core-satellite structure	[36]
	Use of heteroassembled sandwich structure with multiple layers of Au NPs	[59]
	Use of LSPR signal enhancer molecule (e.g., berberine)	[61]
	Combining LSPR and electrochemical sensing	[67]
	Incorporation of enzyme reaction-assisted signal amplification	[63]
	Construction of a 3D nanocup platform	[50]
	Implementation of a split aptamer	[124]
	Integration of microfluidics Incorporation of silver enhancement using catalytic reduction of silver around AuNPs.	[78,116,125] [90]
Low cost, Large scale fabrication	Use of copper	[66]
	Use of PDMS	[50]
	Use of silicon as a substrate Use of plastic optical fiber	[65] [92]
Quantification	Use of double calibration curve method	[40]
Multiple detection	Use of NPs with different sizes and shapes Integration of microfluidic system containing multiple channels	[31] [78]
Real-time detection	Combined system consisting of microfluidic on-chip PCR and LSPRi Use of a microfluidic nanoplasmonic platform	[116]
		[125]
Reproducibility	Fabrication of periodically ordered array using PS with different sizes via the imprinting method	[50]
Reusability	Washing with solution such as PBS or containing SDS Cutting and polishing a tip of optical fiber	[75,79,81] [80]

^a^ Abbreviations: Qdot, quantum dot; NP, nanoparticle; 3D, three-dimensional; PDMS, polydimethylsiloxane; LSPRi, LSPR imaging; PS, polystyrene.

This review discusses a diverse range of approaches that have been proposed for the development of LSPR biosensors. The future development of diverse plasmonic materials, nanostructure architectures, surface modifications, and receptors will play an increasingly critical role in the advancement of LSPR sensing technologies for biomolecule sensing in clinical and environmental settings. Additionally, the fields of nanotechnology and biotechnology will undoubtedly continue to make important breakthroughs toward the development of biosensors.

## Data Availability

No new data were created in this study. Data sharing is not applicable to this article.
